# Early Evaluation of Copper Radioisotope Production at ISOLPHARM

**DOI:** 10.3390/molecules23102437

**Published:** 2018-09-24

**Authors:** Francesca Borgna, Michele Ballan, Chiara Favaretto, Marco Verona, Marianna Tosato, Michele Caeran, Stefano Corradetti, Alberto Andrighetto, Valerio Di Marco, Giovanni Marzaro, Nicola Realdon

**Affiliations:** 1 Legnaro National Laboratories, National Institute of Nuclear Physics, 35020 Legnaro, Italy; francesca.borgna@lnl.infn.it (F.B.); michele.ballan@lnl.infn.it (M.B.); marco.verona@studenti.unipd.it (M.V.); marianna.tosato@studenti.unipd.it (M.T.); michele.caeran@studenti.unipd.it (M.C.); stefano.corradetti@lnl.infn.it (S.C.); alberto.andrighetto@lnl.infn.it (A.A.); valerio.dimarco@unipd.it (V.D.M.); 2 Department of Physics and Earth Science, University of Ferrara, 44122 Ferrara, Italy; 3 Department of Pharmaceutical and Pharmacological Sciences, University of Padua, 35131 Padua, Italy; chiara.favaretto.3@gmail.com; 4 Department of Chemical Sciences, University of Padua, 35131 Padua, Italy

**Keywords:** copper, radionuclide production, FLUKA, Monte Carlo

## Abstract

The ISOLPHARM (ISOL technique for radioPHARMaceuticals) project is dedicated to the development of high purity radiopharmaceuticals exploiting the radionuclides producible with the future Selective Production of Exotic Species (SPES) Isotope Separation On-Line (ISOL) facility at the Legnaro National Laboratories of the Italian National Institute for Nuclear Physics (INFN-LNL). At SPES, a proton beam (up to 70 MeV) extracted from a cyclotron will directly impinge a primary target, where the produced isotopes are released thanks to the high working temperatures (2000 °C), ionized, extracted and accelerated, and finally, after mass separation, only the desired nuclei are collected on a secondary target, free from isotopic contaminants that decrease their specific activity. A case study for such project is the evaluation of the feasibility of the ISOL production of ^64^Cu and ^67^Cu using a zirconium germanide target, currently under development. The producible activities of ^64^Cu and ^67^Cu were calculated by means of the Monte Carlo code FLUKA, whereas dedicated off-line tests with stable beams were performed at LNL to evaluate the capability to ionize and recover isotopically pure copper.

## 1. Introduction

The ISOLPHARM project aims at the production of a wide set of high purity radionuclides for medical use, either for diagnosis or for therapy, according to the Isotope Separation On-Line (ISOL) technique, an accelerator-based method currently being implemented at the Legnaro National Laboratories of the National Institute for Nuclear Physics. In particular, ISOLPHARM is an application of the Selective Production of Exotic Species (SPES) facility [1], which is under advanced construction phase at Legnaro. The ISOLPHARM project will, indeed, exploit the SPES Radioactive Ion Beams (RIBs) to produce high quality and high purity radionuclides in Legnaro that could be used for radiopharmaceutical applications.

Invented almost 70 years ago in Copenhagen, the ISOL technique was further developed at CERN where the ISOLDE facility is currently running [2]. Nowadays other ISOL facilities, already operating or under construction, are disseminated around the world [3,4,5,6]. Thanks to such dissemination of know-how, the ISOL technique was further developed and studied resulting as one of the main techniques for on-line isotope production of high-intensity and high-quality radioactive ion beams [7].

Recently, the ISOL method has been proposed as a promising way to produce exotic radionuclides for medical application. Müller et al. successfully harvested terbium radioisotopes at ISOLDE facility and, after chemical purification, could test them preclinically and, lately, in humans. Based on the promising results, MEDICIS, a new dedicated facility, has been constructed at CERN [8]. The strength of the method is the mass separation, which is performed intrinsically and which allows one to obtain n.c.a. radionuclides.

**Figure 1:**
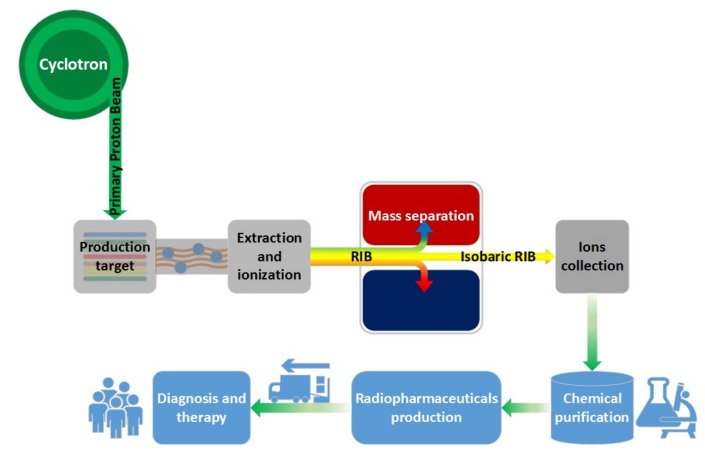
Schematic overview of the ISOLPHARM method. The grey boxes represent the isotopes production according to the ISOL technique, whereas the blue boxes summarize the chemical and pharmaceutical processes.

Briefly, according to the ISOL method (Figure 1), a primary proton beam, extracted from the 70 MeV-750 µA cyclotron recently installed and commissioned in Legnaro, will directly impinge a production target, where, thanks to different nuclear reactions, the radioactive species will be produced. Due to high temperature and high vacuum (~2000 °C and 10^−6^ mbar) the nuclides will be able to diffuse and effuse from the target and thus being ionized (1+) by using one the ion sources depending on the element (SIS, PIS or RILIS) developed for the SPES project [9,10,11,12,13]. After ionization the charged radionuclides will be accelerated (−40 kV) thus producing RIBs. The most important part of the method for ISOLPHARM is the following step, i.e., the mass separation. This step will make possible to have an isobaric beam having the nuclei of the desired atomic mass, thus clearing away all isotopic contaminants, which cannot be chemically eliminated. After mass separation, isotopes are recovered on a secondary target, which is afterwards dissolved in an appropriate medium and purified in case of isobaric contaminants. The final solution can be subsequently used for the radiolabeling of specific molecules, that are finally delivered for preclinical studies and in the future potentially for the clinics both for diagnostic and therapeutic purposes (Figure 1) [14].

The presented method shows many intrinsic advantages:

(1) Extremely high radionuclidic purity can be achieved due to both mass separation and chemical purification. For many nuclides such purity may not be achievable with the traditional techniques (reactors, accelerators) [15].
(2) The isotope production can be very flexible. Indeed, different isotopes of various elements can be produced inside the same production target and by simply adjusting the settings of the mass separator, they can be extracted and recovered independently and subsequently on the collection substrate. This is particularly true when a fissile target is used.
(3) The variety of nuclei producible with the ISOL technique is very wide, thus opening the possibility to easily test exotic radionuclides towards the development of new radiopharmaceuticals. Some of the radionuclides, which will be investigated for production, are: ^43^Sc, ^47^Sc, ^111^Ag and ^149^Tb.
(4) ISOL is an accelerator based technique, thus, with respect to nuclear reactor facilities, it has a lower impact on the environment because of the smaller amount of nuclear wastes produced.

Among the radionuclides that can potentially be produced in the framework of the ISOLPHARM project, we present with in this work the preliminary results of the experiments carried out for copper. This element has been selected since ^64^Cu and ^67^Cu are a promising theragnostic pair [16]. 

^64^Cu can be used both for diagnostic (PET) and therapeutic use, thanks to its co-emission of β^+^ and β^−^ particles (t_1/2_ = 12.701 h) and is currently under preclinical and clinical development [17]. It is commonly produced with medical cyclotrons impinging a proton beam on a nickel target via or the preferable ^64^Ni(p,n)^64^Cu reaction, or the ^64^Zn(d,2p)^64^Cu reaction when less activity is required [18].

^67^Cu is a β^−^ emitter (t_1/2_ = 2.58 d, Eβ^−^_av_ 162 keV) ideal for therapeutic applications. Its low-energy gamma co-emission (185 keV, intensity 48.7%) makes it suitable for SPECT applications as well. Moreover it represents a perfect matched pair for theragnosis with the PET isotope ^64^Cu, enabling the diagnosis and the subsequent therapy using chemically identical molecules [16]. Despite its highly potential use for the clinics, its use is nowadays very limited, due to shortage in the production. Müller et al. recently stated the need of putting further efforts in the production of this radionuclide, thus enabling the assessment of its therapeutic potential [16].

The ISOL technique could have the potential to simultaneously produce decent amounts of ^67^Cu with ^64^Cu, using the same production target, but thanks to mass separation it would be possible to recover them independently.

As a proof of principle, in this study we first evaluated a possible ISOL production target for both ^64^Cu and ^67^Cu. Zirconium germanide (ZrGe), an innovative material under development in our labs, was identified as a production target and the expected yields are described in this paper. Such results were obtained using the Monte Carlo code FLUKA [19].

The second part of this work aimed to assess the feasibility of producing copper ion beams in Legnaro. The ISOL RIB production, indeed, includes several steps, which have to be quantitatively characterized in order to estimate the real final output. The method can be roughly split as follows:

(1) Release of the produced nuclei from the target (diffusion through the solid matrix and effusion through the pores);
(2) Ionization and extraction of the beam;
(3) Mass separation;
(4) Recovery of the ion beam.

Thanks to the use of the SPES Front End (FE) [12] in off-line modality, so not connected to the proton beam, it was possible to investigate the steps from 2 to 4. Ionization was performed with the Mass Marker system, for the mass separation the SPES Wien Filter (WF) was used and, finally, copper beams were recovered on sodium chloride secondary targets, as previously done for strontium and yttrium in our group [14].

## 2. Results

### 2.1. Numerical Evaluation of the Production of ^64^Cu and ^67^Cu with the ISOL Technique

Taking into account the available proton beam energy at SPES (up to 70 MeV) and the requirements for the ISOL targets (the target has to operate at very high temperature without significant deterioration), Zirconium germanide was considered as a new possible target material to produce ^64^Cu and ^67^Cu in the framework of ISOLPHARM. Indeed, such material was stated as suitable for the ISOL technique, because, being refractory, it can be employed in the expected working temperature ranges (up to 1800 °C) [20,21,22]. Such high temperatures are in fact required to boost the release of the in-target produced copper isotopes, fundamental step for the creation of the RIB. In addition, according to TENDL2015 [23], the ^nat^Ge(p,X)^64^Cu and ^nat^Ge(p,X)^67^Cu show acceptable cross sections for the SPES proton energy range. Thanks to the MC code FLUKA we could calculate the in-target production. For ^64^Cu the yield after 5 days of irradiation with a proton beam of 100 µA and 70 MeV was 55.2 GBq, while for ^67^Cu was 1.4 GBq (Table 1). Other proton beam energies and intensities, together with different irradiation times, were also tested and the results are reported in the Supporting Information (Tables S1–S4).

In addition, we could verify that other interesting radioisotopes could be produced with this target material, such as ^72^As (t_1/2_ = 26 h, β^+^ emission), ^74^As (t_1/2_ = 17.7 d, β^−^ Eβ′_average_ = 242.75 keV, 15.4% and Eβ′′_average_ 1352.8, keV 19.0%; β^+^ Eβ^+^_average_ = 408.0 keV, 26.1%) and ^77^As (t_1/2_ = 38.79 h, β^−^ Eβ^−^_average_ 228.8 keV, 97%).

The reliability of such numerical results is currently under investigation considering different theoretical models (TALYS, EMPIRE) for the calculation of the excitation functions for the nuclear reactions taken into account in this study.

The results in Table 1 are the theoretical activities in the production target, but such values have to be corrected by the efficiency factors related to the subsequent release, ionization, mass separation and recovery processes, as later discussed.

### 2.2. Ionization of Copper and its Efficiency

In order to evaluate the capability of the SPES FE to efficiently ionize copper isotopes and extract them into a beam, ionization tests were performed by loading precise amounts of stable copper (^63^Cu 69.17%, ^65^Cu 30.83%) into the ion source. Such tests were performed using the SPES FE in off-line modality, namely without the proton beam and the production target. Copper was introduced inside the FE by means of the Mass Marker (MM) technique [14]. Such method foresees the surface deposition of a small amount of the desired element (generally 40 μg) on a thin tantalum foil (the Mass Marker—MM), that is lately accurately folded and inserted inside a small tubular oven, that replaces the production target. Such oven can be heated by Joule effect, allowing the atomization of the substrate previously deposed on the foil, and the migration of the neutrals towards the ion source. In the case of copper the SPES Plasma Ions Source (PIS) was used [9]. ^nat^Copper ionization occurred after heating the oven, using currents from 30 A to 80 A, with 5 A steps, thus working in a temperature range of 1200–2000 °C. The stepwise increase of the heating current, allowed to gradually release the loaded copper isotopes towards the ion source. In addition to the heating, the use of the plasma ion source and of the acceleration devices of the SPES FE allowed the detection of an ion beam in the Faraday Cup 2 (FC2, see FE description). The copper current measured was between the 200 and 600 nA.

**Figure 2:**
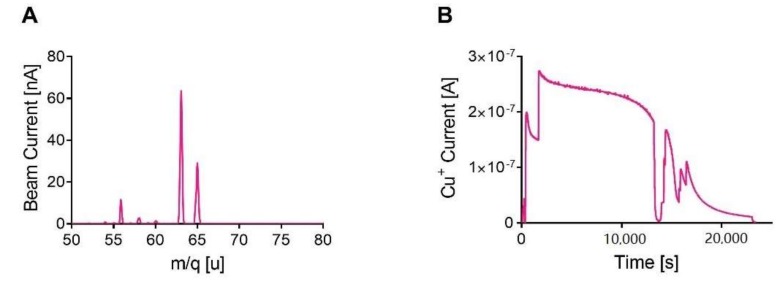
(**A**) Mass scan of copper beams and (**B**) trend of copper beam in time during ionization tests.

We could verify ^nat^copper ionization thanks to the analysis of masses composing the beam. A typical mass scan for copper is reported in Figure 2A. Copper is clearly identified thanks to the two peaks of masses 63 and 65. The isotopic abundance ratio was checked by the comparison of the peaks’ heights, which gave a value of 68.7/31.3, very close to expected values of 69.2/30.8.

The ionization efficiency, i.e., the total amount of ^nat^copper ionized out of the amount of ^nat^copper in the MM, was calculated repeating the assay every time with a new MM until all the copper was evaporated and no more copper current could be detected. The time necessary was around 4 h keeping the Cu^+^ current at about 200 nA, giving an efficiency of 7.67 ± 1.3%. In Figure 2B the copper beam current trend is reported. For the ionization efficiency calculation, three independent tests were performed.

### 2.3. Deposition of Copper on Sodium Chloride Disks and its Recovery

**Figure 3:**
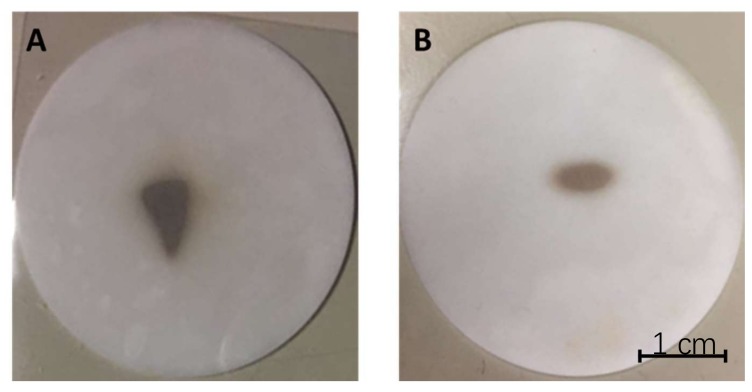
The copper beam spots on the sodium chloride discs after the July deposition tests (**A**) and September (**B**).

To prove that copper could be recovered from the beam, a solid target (called secondary target) was placed at the end of the beam line and, after the target removal from the beam line and dissolution, copper was quantified. In particular, ^nat^copper beams were recovered by placing, as a secondary target, a sodium chloride disk that was interceptive with the beam. The sodium chloride disks were prepared as previously reported [14]. ^nat^Copper beams were produced with the SPES FE as described for the ionization tests and PIS was used. After the removal of the secondary target at the end of several hours of irradiation, during which the copper beam was impinging on the NaCl disk, the appearance of the disk was as reported in Figure 3A,B. 

All the secondary targets displayed a clear brownish spot in the middle of the disk. The spots were attributed to a copper surface deposition and not to thermal degradation of the material, due to the very low energy of the extracted beam (25 keV). As described in Section 4.3, a total of five tests were carried out. In the first four tests only ^63^Cu was recovered on the secondary target by setting the mass separator on mass 63 and thus deviating mass 65 out of the secondary target and resulting in only one spot on them, see Figure 3A,B. The first two recovery tests, carried out with the FE at the upgrade level of July 2017, had a spot like that reported in Figure 3A, while the latest, performed in September 2017 had a smaller spot (Figure 3B).

**Figure 4:**
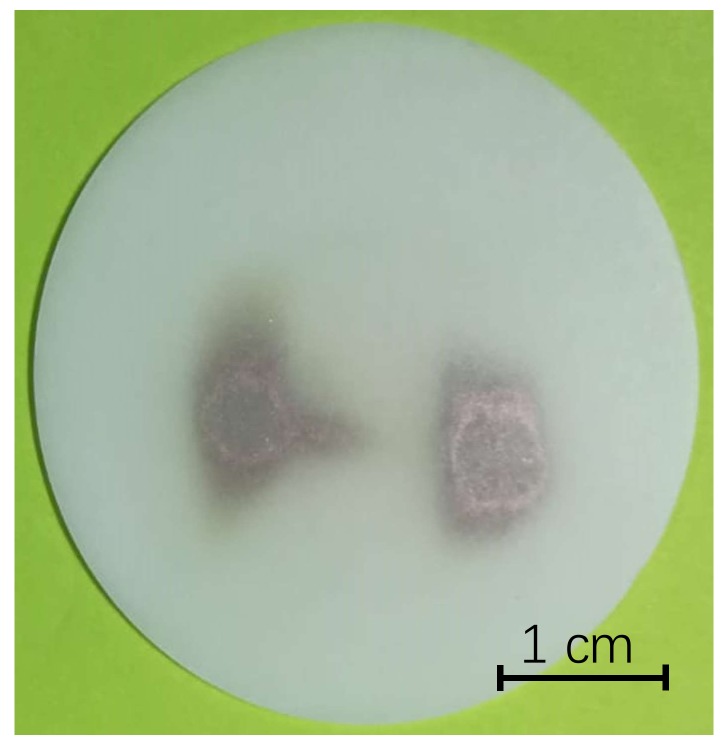
The ^63^Cu and ^65^Cu spots on the secondary target from right to left.

To prove the possibility of producing at the same time ^64^Cu and ^67^Cu when the facility will be operating on-line, we carried out one more recovery test where we recovered both masses of ^nat^Cu, ^63^Cu and ^65^Cu, on the same secondary target. As visible in Figure 4, it was possible to recover both as two clearly separated spots.

All the secondary targets used for copper recovery were used for copper quantification via Graphite Furnace-Atomic Absorption Spectrometry (GF-AAS) after dissolution in a proper medium, see Table 2. These data were compared to the total amount of copper impinging the secondary target, which were calculated measuring the copper current at regular time points and by integrating the beam current.

For the first two recovery tests the NaCl disks were irradiated for a longer time and with higher beam currents. The calculated amount of copper impinging the target was 9.94 and 5.21 µg, respectively. After dissolution in 0.5 M HNO_3_, we could quantify the copper present on the disk, which turned out to be 1.46 and 1.09 µg, respectively. During depositions 3 and 4, 1.12 and 0.94 µg were calculated on the target, while the measured ones via GF-AAS were 0.54 and 0.50 mg, respectively. The last irradiated sodium chloride target, Figure 4, was split into two in order to analyze the ^63^Cu and ^65^Cu independently. The ^63^Cu spot was 4.72 µg, while ^65^Cu was 1.98 µg, the 70.5 and 29.5% of the total amount of copper recovered respectively, thus respecting the natural isotopic natural abundance of copper.

## 3. Discussion

In this study we performed a proof of principle study to evaluate the possibility of producing ^64^Cu and ^67^Cu at SPES facility at the Legnaro National Laboratories of the Italian National Institute for Nuclear Physics (INFN-LNL) when the ISOL facility will be running.

The possible production yields of the aforementioned isotopes were calculated by means of the Monte Carlo code FLUKA, showing promising results. The proton beam energy of 70 MeV (maximum proton beam energy available at LNL) was selected to maximize the yields by dumping the entire beam in the target. As a consequence, even if the cross section peaks of the reactions ^nat^Ge(p,X) ^64^Cu and ^nat^Ge(p,X) ^67^Cu are expected to be low in the range 0–70 MeV [24], the production of ^64^Cu and ^67^Cu is still sufficient thanks to the overall integrated cross section in such energy range: ~55 GBq and 1.4 GBq respectively in the production target after 5 days of irradiation. However, no experimental data were found for such reactions, consequently future irradiation tests will be performed to determine the cross sections and verify the presented results. In addition, different models for the calculation of the excitation functions of nuclear reactions are being considered (TALYS, EMPIRE) in order to cross check the FLUKA results. Anyway the ISOL technique involves several steps and each of them affects the process efficiency. Consequently, in the secondary target we expect to collect significantly lower quantities. This drawback can make the ISOL production of copper isotopes less competitive than cyclotron-based techniques, which provide amounts of ^64^Cu with few hours of irradiation [18]. On the contrary ^67^Cu is currently very difficult to have in significant quantities for preclinical studies, thus ISOL could represent a valid option to satisfy this need. In any case the strength point of this study is the possibility to simultaneously produce both of them, but with the capability to recover them independently and making this way the joint study of both radioisotopes significant.

As previously stated, the different processes involved in ISOL have their own efficiencies. The offline ionization tests performed in this study were aimed to quantify the influence of the ionization, the extraction, the mass separation and recovery on the overall production. It was possible to verify that intense (up to 600 nA) ^nat^copper ion beams could be produced using the Plasma Ion Source and the MM technique. Indeed, copper was easily and steadily released when the MM was heated by Joule effect [9], showing a good volatility even at low temperatures (1200 °C). The mass separation step was also verified with these experiments. Indeed, we used the SPES Wien Filter, which will be installed for the on-line facility. We could clearly separate the masses 63 and 65, as shown in Figure 2A and Figure 4, thus enabling us to extend these results to masses 64 and 67, being in the order of magnitude of mass. According to such tests we measured an ionization efficiency factor of ~8 %. The presented result could be potentially increased by switching to the resonant laser ionization technique [13], that normally ensures a more selective ionization, since the laser is tuned to the typical wavelengths of the desired element [25]. Dedicated tests with a laser ion source could be performed in the future to identify the most efficient ionization mechanism for copper.

In this study we could not quantify the efficiency of the processes involved in the release of copper from the ZrGe production target. Such measurement is the trickiest part, since the release includes the diffusion and the effusion processes in the ZrGe matrix. Preliminary experiments to determine the effusion are currently being performed. Regarding the diffusion, a direct experimental measurement will not be possible in the offline modality, but a customized Monte Carlo numerical model is currently under development in our group to predict it.

Finally, copper ion beams could be recovered thanks to the use of a low-cost and low-toxic material: sodium chloride. These substrates, previously developed for the recovery of other ion beams [14], could be easily produced by direct compression of sodium chloride powder and proved to be stable and resistant during the irradiation time. Moreover, the use of ultrapure starting material avoids the introduction of metallic impurities, which are highly undesired in the production of radiometals for nuclear medicine. Thanks to the white color of the disks the copper beam spots could be clearly visualized, see Figure 3 and Figure 4. The quantification of copper after the recovery from the beam was made possible thanks to the dissolution of the targets in acidic medium. When diluted nitric acid was used, only a low amount of copper was measured, so the dissolution in concentrated nitric acid and at high temperatures was then applied. In this case the amount of copper recovered was higher, but still not the 100% of the copper which was foreseen on the targets. For this reason, further studies will be carried out to improve the efficiency of the chemical recovery process, even if part of the problem might be due to the distance between the position of the sodium chloride targets and the FC2, the device used for the monitoring of the copper beams. In the future purification processes for the elimination of isobaric and pseudo-isobaric contaminants will be developed.

As a final consideration, this study was a proof of principle for the future production of copper isotopes in Legnaro at the SPES facility. Such a facility will have the possibility to produce a wide range of nuclei, so similar experimental activities can be performed for each of the most relevant radionuclides with possible medical application. One limit of SPES facility is the maximum available proton energy (70 MeV), which limits the possible nuclear reaction types, consequently the variety of elements producible with one target is generally narrow. This is particularly true in the case of neutron-poor nuclides, or light nuclei: the target material should include stable nuclei close in mass to the desired radionuclide. However, in the case of neutron-rich species, fissile targets as Uranium Carbide can be used, thus generating with one target a wide range of nuclides. Dedicated targets are designed only in the case the fission production of the nuclide of interest is not possible or too low, as in the case of the pair ^64^Cu and ^67^Cu.

Generally speaking, we can consider ISOLPHARM as a test bench for evaluating the capability to develop new carrier-free radionuclides exploiting the nuclei producible with the ISOL technique. Indeed, as one of the application of SPES facility, ISOLPHARM beam time will be limited by the scheduled physics program. This will not be an issue, since for preclinical studies only little amounts of radionuclides are necessary, thus, for example, the irradiation during the weekends could be enough. Once the concept is proven, other higher energy ISOL facilities may be interested in implementing the method and the know-how developed in Legnaro.

## 4. Materials and Methods

### 4.1. Monte Carlo Simulations for the ^64^Cu and ^67^Cu Production Evaluation

FLUKA (2011.2c developed by CERN-INFN) is a fully integrated particle physics Monte Carlo simulation software developed by INFN and CERN, used for simulating the interaction between particles and matter [19]. Such code was used in the framework of this study for the calculation of the produced activities of ^64^Cu and ^67^Cu. A simplified cylindrical geometry was considered for the Zirconium germanide target with a 4 cm diameter and an estimated material density of 4 g/cm^3^. A target thickness of 5cm was used, to ensuring the dumping of the beam within the production target. For the 70 MeV 100 µA (6.242E + 14 p/s) a Gaussian profile was used with rms of 0.5 cm in both transverse axes. Radioactive decay chains were activated, meaning that, in addition to the physical yields, the calculated activities take into account both the additive contribution of the decayed parent nuclides and the subtractive amount of decayed nuclei of interest. Different irradiation times were considered, in particular 0.5, 1, 2, 3, 4, 5, 6, 7, 8, 10, 15 days, typical irradiation times for ISOL targets at SPES (the maximum planned time is 15 days). The RESNUCLEi card was used for the scoring of the residual nuclei production in the target. The obtained results were consequently corrected by an ISOL technique efficiency factor, obtained by multiplying a first coefficient that takes into account the ionization processes efficiency and a second one related to the mass separation-beam transport processes. In the case of copper, the experimentally determined value of 7.67% was considered for the ionization efficiency, as estimated with the dedicated off-line tests, whereas a reasonable factor of 90% was hypothesized for the latter processes.

### 4.2. ^nat^Copper Ionization and Beam Production

**Figure 5:**
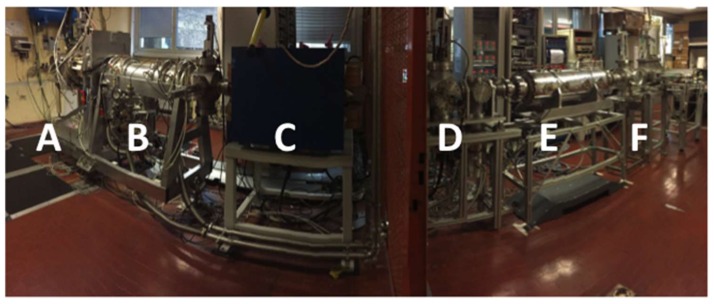
The SPES Front End in off-line modality. (**A**) Target and ion source unit; (**B**) First triplet and steers unit and diagnostic device; (**C**) Mass separator, Wien Filter; (**D**) Second diagnostic unit; (**E**) Second triplet system; (**F**) Secondary target for beams recovery station.

Stable copper beams were produced using the experimental apparatus developed for the SPES project, namely the SPES Front End (FE). This apparatus will be connected to the primary proton beam for the RIBs production, after being moved into the bunker of the SPES building and is now used as test bench for the production of stable ion beams in off-line modality, allowing this way the R&D development of the ion sources, of the mass separators and of the diagnostic devices. A comprehensive description of the FE is reported in the literature [14] and a picture is shown in Figure 5.

Briefly, it is made of six main functional subsystems: the target and ion source unit (Figure 5A), the first beam optics subsystem and the first diagnostic box (Figure 5B), the Wien filter (Figure 5C), the diagnostic box 2 (Figure 5D), the second beam optic subsystem (Figure 5E) and the secondary target for beams recovery (Figure 5F). The ion source complex is placed inside a vacuum chamber that enables the use of the ion source at high temperatures with pressure levels between 10^−5^ and 10^−6^ mbar. In off-line mode, no primary target is installed inside the chamber, but different methodologies are used to introduce the stable isotopes to be ionized and accelerated depending on the physical state of the element. In the case of gases, they are introduced through a controlled gas flow and injected in the ion source by means of a calibrated leak; in case the element to be ionized is solid, it is usually chosen in the form of a soluble salt, dissolved in acidic media whit a precise concentration (usually around 1 g/L). A drop of such solution can be quantitatively deposed on a tantalum foil, called Mass Marker (MM, CsC, Schio, Vicenza, purity higher than 99%) and the solvent is lately evaporated, obtaining thus a surface deposition of the chosen salt on the foil. The MM is then carefully folded [14] and inserted into tantalum tube, called oven, that can be heated by Joule effect, thus allowing the element atomization and injection into the ion source.

The ion sources developed for the SPES project are of two kinds, depending on the first ionization potential of the element. For elements of the 1st and 2nd group, the Surface Ion Source (SIS) is adopted [9,10]; for those with higher electronegativity, the Plasma Ion Source (PIS) is required [11,12].

The ion source used in these tests is the SPES PIS, which is a forced electron beam induced arc discharge ion source. It is a non-selective device capable of ionizing a large spectrum of elements, mainly composed of two parts: the tantalum cathode and the molybdenum anode. The former is heated at 2200 °C by the Joule effect, generating an intense thermionic emission of electrons (free electrons) on the cathode surface facing the anode. The anode, at 150 V with respect to the rest of the ion source, attracts the ionizing electrons, in this way allowing the creation of a plasma inside its cylindrical cavity [11,12].

The ion source is placed on a 25 kV platform with respect to the extraction electrode at ground potential. The high voltage can however be increased to 40 kV. The beam optics subsystem is made of a set of electrostatic deflectors and a quadrupole triplet which allow beam alignment and focusing, respectively. In each diagnostic box there is a Faraday Cup (FC) for beam intensity monitoring and a grid-based beam profile (BP) detector. 

The beam mass selection is performed by a Wien Filter (WF), a device which is able to select a specific ion due to mutually perpendicular electric and magnetic fields orthogonal to the ion velocity. The undeflected particles have speed equal to the ratio between electric and magnetic field. The deflected ions are then stopped by a slits subsystem. Since the various masses have approximately the same energy (25 keV), this device can be used in the FE as a mass selector. For this reason, the Wien Filter is composed by a vacuum chamber where two electrodes, maintained at a certain potential, provide the desired electric field and by a magnet excited by two coils, that supplies the magnetic field. Following mass separation, a second diagnostic box is installed, constituted by a FC, a grid-based BP detector and an emittance meter device.

For copper beams production and ionization efficiency determination, the Mass Marker (MM) and oven systems were used coupled to the SPES PIS. 40 µL of copper standard solution (1 g/L, FLUKA Analytics) were dropped on a 10 × 10 mm tantalum foil and the MM, after being folded, was inserted into the oven at 80 mm distance from the opening. The oven was then closed on one side and connected to the transfer line on the other one. The following parameters were set for the FE operation: transfer line current 415 A, oven maximum current 80 A, extractor position 50%, S1, S2, S3 and S4 0 V and Q1, Q2 and Q3 1395, 695 and 1398 V. Natural copper is an isotopic mixture: ^63^Cu (69.17%) and ^65^Cu (30.83%). For all the ionization tests and the first recovery tests the most abundant isotope was considered. For the isolation of mass 63, the mass separator, which is a Wien Filter, was used (V_p_ 2579, V_pe_ 590 V^1^ and I_WF_ 111 A) and the slits were open (18 mm). The ^63^Cu beam current was measured until no more current could be detected. The current was integrated in time using the software Origin Pro 2015 to determine the total amount of copper ionized. The ionization efficiency was calculated as a percentage of the initial amount of copper dropped in the MM.

### 4.3. ^nat^Copper Beam Recovery

Copper beams were recovered placing a sodium chloride (1.8 g) secondary target at the end of the beam line (see Figure 5F). Prior to each deposition, one MM loaded with 200 µL of copper nitrate (1 g/L) was inserted into the oven (from 4 to 8 cm from the oven closure) and connected to the PIS transfer line. For the first recovery tests where ^63^Cu was recovered the slits were open at 18 mm, and the triplets and steerers set as follow: Q1, Q3 and Q3 875, 695 and 1398 V and S1, S2, S3 and S4 0 V. The WF was switched on Vp 2579, Vpe 590 V and IWF ~111 A. The IWF was checked prior to each test, closing the slits at 2 mm in order to allow the best mass selection (^63^Cu). The second triplet subsystem was set as follow: QT1, QT2 and QT3 1760, 850 and 1775 V, respectively. The best focalization was possible thanks to the use of the BP3.

The oven was then switched on, the FC3 with the NaCl disk built on it was put into the channel, while the FC2 and all the other interceptive devices were removed from the channel, to allow the copper beam to impinge on the secondary target. The oven was heated up to a maximum of 65 A, and the deposition carried out for 4–5 h. The FC2 was used to detect the ^63^Cu current every 20–30 min. The values of current were integrated in time using the software Origin Pro 2015 to estimate the total copper atoms reaching the NaCl target.

After the recovery, the target was removed and dissolved for analysis. Prior to dissolution, since the beam spot was very clear and to reduce the sodium chloride amount, the portion of the disk where the copper was visible was separated from the rest, breaking the substrate. Two different procedures were followed for copper extraction from the disk. In the first 2 depositions, the disk was dissolved in 20 mL of HNO_3_ 0.5 M, under stirring and mild heating. After 20 min the solution was quantitatively transferred to a 100 mL flask and the volume adjusted with HNO_3_ 0.5 M. The 3rd and 4th disks were treated differently; after weighing, they were transferred to a Teflon vessel, and 6 mL of concentrated HNO_3_ were added. The vessels were then closed and heated up thanks to a microwave oven to 180 °C for 20 min. After cooling down, the solutions were transferred to two 50 mL-flasks and the volume adjusted to 50 mL. In both cases standards at 100 µg/L were prepared for Graphite Furnace Atomic Absorption Spectroscopy (GF-AAS, Varian, Palo Alto, CA, USA; lamp current = 4 mA, λ = 327.4 nm).

## 5. Conclusions

In conclusion, with this proof-of-concept study we evaluated the capability to produce copper isotopes with the ISOL technique in Legnaro. Zirconium germanide looks as a promising material for ISOL application and is currently under characterization at the Legnaro laboratories. The preliminary calculations using the Monte Carlo code FLUKA suggested that using the SPES 70 MeV cyclotron significant amounts of ^64^Cu and ^67^Cu can be produced. The reliability of such results is currently under investigation with different MC codes. The capability of SPES Front End to ionize stable copper atoms was tested and copper ion beams were recovered on sodium chloride targets. As a matter of fact, this study is not intended to limit the scenario of radionuclides production at ISOLPHARM to copper though, being a proof of principle of the technique, such work may pioneer the possibility to study the ISOL production of other radionuclides of medical interest.

## Figures and Tables

**Table 1:** FLUKA calculated in-target activities for ^64^Cu and ^67^Cu nuclides, produced in five days irradiation of the ZrGe target with a 100 μA 70 MeV proton beam.

Isotope	FLUKA Calculated Activity (5 Days Irradiation with Proton Beam)
^64^Cu	55.2 GBq
^67^Cu	1.4 GBq

**Table 2:** Results of the tests for copper recovery. The values reported in the first column are the expected amount of copper impinging the secondary target, calculated by integrating the copper beam current in time (measures taken at regular time points, not continuously). In the second column the amount of copper recovered by each target is reported. The quantification was made possible thanks to the target dissolution (see third column) and analysis (GF-AAS).

	Copper (Current) Measured in FC2 and Integrated in Time [µg]	Copper Measured via GF-AAS [µg]	
1st deposition	9.94	1.46	Target dissolved in HNO_3_ 0.5 M, mild heating
2nd deposition	5.21	1.09	Target dissolved in HNO_3_ 0.5 M, mild heating
3rd deposition	1.12	0.54	Target dissolved in concentrated HNO_3_, 180 °C for 20 min
4th deposition	0.94	0.50	Target dissolved in concentrated HNO_3_, 180 °C for 20 min

## Supplementary Materials

The following are available online, Table S1: FLUKA calculated activities for ^67^Cu nuclides, produced in five days of irradiation of the ZrGe target with a 40 MeV proton beam, Table S2: FLUKA calculated activities for ^67^Cu nuclides, produced in five days of irradiation of the ZrGe target with a 70 MeV proton beam, Table S3: FLUKA calculated activities for ^64^Cu nuclides, produced in five days of irradiation of the ZrGe target with a 40 MeV proton beam, Table S4: FLUKA calculated activities for ^64^Cu nuclides, produced in five days of irradiation of the ZrGe target with a 70 MeV proton beam.
