# Supplementary material for: Early Evaluation of Copper Radioisotope Production at ISOLPHARM
Source: Molecules. 2018 Sep 24;23(10):2437. doi: 10.3390/molecules23102437 (PMC6222311; doi:10.3390/molecules23102437)
Supplement: Supplementary file 1 [file molecules-23-02437-s001.pdf]

Supporting Information

Early evaluation of copper radioisotope production at ISOLPHARM

F. Borgna [1,+], M. Ballan [1,2,+], C. Favaretto [3], M. Verona [1,3], M. Tosato [1,4], M. Caeran [1,3], S. Corradetti [1], A. Andrighetto [1], V. Di Marco [1,4], G. Marzaro [1,3,*], N. Realdon [3,*]

1 Legnaro National Laboratories, National Institute of Nuclear Physics, 35020 Legnaro, Italy

2 Department of Physics and Earth Science, University of Ferrara, 44122 Ferrara, Italy

3 Department of Pharmaceutical and Pharmacological Sciences, University of Padova, 35131 Padova, Italy

4 Department of Chemical Sciences, University of Padova, 35131, Padova, Italy

* Correspondence: giovanni.marzaro@unipd.it (G.M.); Tel: +39-049-827-5024 (G.M.); nicola.realdon@unipd.it (N.R.); Tel: +39-049-827-5338 (N.R.)

\+ The authors equally contributed to the experiments and to the writing of the manuscript and should be considered as co-first authors

Numerical evaluation of the production of ^{64}Cu and ^{67}Cu with the ISOL technique

The production yields (ionization and transport corrected) for ^{64}Cu and ^{67}Cu were evaluated, according to the methods reported in the main manuscript, at two beam energies (40 and 70 MeV) and four beam intensities (5, 10, 50 and 100 μA). The full results are presented in tables S1, S2, S3 and S4.

Table S1: FLUKA calculated activities for ^{67}Cu nuclides, produced in five days of irradiation of the ZrGe target with a 40 MeV proton beam.

^{67}Cu production ($t_{1/2}$: 61,83 h)								
40 MeV								
	5 μA		10 μA		50 μA		100 μA	
Time	Activity		Activity		Activity		Activity	
[days]	[Bq]	[mCi]	[Bq]	[mCi]	[Bq]	[mCi]	[Bq]	[mCi]
0,5	9,05E+04	2,45E-03	1,81E+05	4,89E-03	9,05E+05	2,45E-02	1,81E+06	4,89E-02
1	1,70E+05	4,58E-03	3,39E+05	9,17E-03	1,70E+06	4,58E-02	3,39E+06	9,17E-02
1,5	2,39E+05	6,45E-03	4,78E+05	1,29E-02	2,39E+06	6,45E-02	4,78E+06	1,29E-01
2	2,99E+05	8,09E-03	5,98E+05	1,62E-02	2,99E+06	8,09E-02	5,98E+06	1,62E-01
3	3,98E+05	1,08E-02	7,97E+05	2,15E-02	3,98E+06	1,08E-01	7,97E+06	2,15E-01
4	4,74E+05	1,28E-02	9,48E+05	2,56E-02	4,74E+06	1,28E-01	9,48E+06	2,56E-01
5	5,32E+05	1,44E-02	1,06E+06	2,87E-02	5,32E+06	1,44E-01	1,06E+07	2,87E-01
6	5,76E+05	1,56E-02	1,15E+06	3,11E-02	5,76E+06	1,56E-01	1,15E+07	3,11E-01

Table S2: FLUKA calculated activities for ^{67}Cu nuclides, produced in five days of irradiation of the ZrGe target with a 70 MeV proton beam.

^{67}Cu production ($t_{1/2}$: 61,83 h)								
70 MeV								
	5 μA		10 μA		50 μA		100 μA	
Time	Activity		Activity		Activity		Activity	
[days]	[Bq]	[mCi]	[Bq]	[mCi]	[Bq]	[mCi]	[Bq]	[mCi]
0,5	1,06E+06	2,88E-02	2,13E+06	5,76E-02	1,06E+07	2,88E-01	2,13E+07	5,76E-01
1	2,00E+06	5,39E-02	3,99E+06	1,08E-01	2,00E+07	5,39E-01	3,99E+07	1,08E+00
1,5	2,81E+06	7,59E-02	5,62E+06	1,52E-01	2,81E+07	7,59E-01	5,62E+07	1,52E+00
2	3,52E+06	9,52E-02	7,04E+06	1,90E-01	3,52E+07	9,52E-01	7,04E+07	1,90E+00
3	4,69E+06	1,27E-01	9,37E+06	2,53E-01	4,69E+07	1,27E+00	9,37E+07	2,53E+00
4	5,58E+06	1,51E-01	1,12E+07	3,01E-01	5,58E+07	1,51E+00	1,12E+08	3,01E+00
5	6,26E+06	1,69E-01	1,25E+07	3,38E-01	6,26E+07	1,69E+00	1,25E+08	3,38E+00
6	6,78E+06	1,83E-01	1,36E+07	3,66E-01	6,78E+07	1,83E+00	1,36E+08	3,66E+00

Table S3: FLUKA calculated activities for ^{64}Cu nuclides, produced in five days of irradiation of the ZrGe target with a 40 MeV proton beam.

^{64}Cu production ($t_{1/2}$: 12,7 h)								
40 MeV								
	5 µA		10 µA		50 µA		100 µA	
Time	Activity		Activity		Activity		Activity	
[days]	[Bq]	[mCi]	[Bq]	[mCi]	[Bq]	[mCi]	[Bq]	[mCi]
0,5	1,34E+07	3,61E-04	2,67E+07	7,22E-04	1,34E+08	3,61E-03	2,67E+08	7,22E-03
1	2,03E+07	5,49E-04	4,06E+07	1,10E-03	2,03E+08	5,49E-03	4,06E+08	1,10E-02
1,5	2,39E+07	6,46E-04	4,78E+07	1,29E-03	2,39E+08	6,46E-03	4,78E+08	1,29E-02
2	2,58E+07	6,97E-04	5,15E+07	1,39E-03	2,58E+08	6,97E-03	5,15E+08	1,39E-02
3	2,73E+07	7,37E-04	5,45E+07	1,47E-03	2,73E+08	7,37E-03	5,45E+08	1,47E-02
4	2,76E+07	7,47E-04	5,53E+07	1,49E-03	2,76E+08	7,47E-03	5,53E+08	1,49E-02
5	2,78E+07	7,50E-04	5,55E+07	1,50E-03	2,78E+08	7,50E-03	5,55E+08	1,50E-02
6	2,78E+07	7,51E-04	5,56E+07	1,50E-03	2,78E+08	7,51E-03	5,56E+08	1,50E-02

Table S4: FLUKA calculated activities for ^{64}Cu nuclides, produced in five days of irradiation of the ZrGe target with a 70 MeV proton beam.

^{64}Cu production ($t_{1/2}$: 12,7 h)								
70 MeV								
	5 µA		10 µA		50 µA		100 µA	
Time	Activity		Activity		Activity		Activity	
[days]	[Bq]	[mCi]	[Bq]	[mCi]	[Bq]	[mCi]	[Bq]	[mCi]
0,5	1,19E+08	3,23E-03	2,39E+08	6,46E-03	1,19E+09	3,23E-02	2,39E+09	6,46E-02
1	1,82E+08	4,91E-03	3,63E+08	9,81E-03	1,82E+09	4,91E-02	3,63E+09	9,81E-02
1,5	2,14E+08	5,78E-03	4,28E+08	1,16E-02	2,14E+09	5,78E-02	4,28E+09	1,16E-01
2	2,31E+08	6,23E-03	4,61E+08	1,25E-02	2,31E+09	6,23E-02	4,61E+09	1,25E-01
3	2,44E+08	6,59E-03	4,88E+08	1,32E-02	2,44E+09	6,59E-02	4,88E+09	1,32E-01
4	2,47E+08	6,68E-03	4,95E+08	1,34E-02	2,47E+09	6,68E-02	4,95E+09	1,34E-01
5	2,48E+08	6,71E-03	4,97E+08	1,34E-02	2,48E+09	6,71E-02	4,97E+09	1,34E-01
6	2,49E+08	6,72E-03	4,97E+08	1,34E-02	2,49E+09	6,72E-02	4,97E+09	1,34E-01
